# A Robust and Accurate Indoor Localization Using Learning-Based Fusion of Wi-Fi RTT and RSSI

**DOI:** 10.3390/s22072700

**Published:** 2022-03-31

**Authors:** Hamada Rizk, Ahmed Elmogy, Hirozumi Yamaguchi

**Affiliations:** 1 Computers & Control Engineering Deptartment, Tanta University, Tanta 31527, Egypt; hamada_rizk@f-eng.tanta.edu.eg; 2 Graduate School of Information Science and Technology, Osaka University, Suita 565-0871, Japan; h-yamagu@ist.osaka-u.ac.jp; 3 Faculty of Computer Engineering & Sciences, Prince Sattam Ibn Abdelaziz University, Al-Kharj 16273, Saudi Arabia; 4 Faculty of Engineering, Tanta University, Tanta 31527, Egypt

**Keywords:** indoor localization, deep learning, fingerprinting, round-trip time, canonical correlation analysis

## Abstract

Great attention has been paid to indoor localization due to its wide range of associated applications and services. Fingerprinting and time-based localization techniques are among the most popular approaches in the field due to their promising performance. However, fingerprinting techniques usually suffer from signal fluctuations and interference, which yields unstable localization performance. On the other hand, the accuracy of time-based techniques is highly affected by multipath propagation errors and non-line-of-sight transmissions. To combat these challenges, this paper presents a hybrid deep-learning-based indoor localization system called *RRLoc* which fuses fingerprinting and time-based techniques with a view of combining their advantages. *RRLoc* leverages a novel approach for fusing received signal strength indication (RSSI) and round-trip time (RTT) measurements and extracting high-level features using deep canonical correlation analysis. The extracted features are then used in training a localization model for facilitating the location estimation process. Different modules are incorporated to improve the deep model’s generalization against overtraining and noise. The experimental results obtained at two different indoor environments show that *RRLoc* improves localization accuracy by at least 267% and 496% compared to the state-of-the-art fingerprinting and ranging-based-multilateration techniques, respectively.

## 1. Introduction

User location becomes one of the most valuable contexts in human-centric environments. This context can be used to enhance a wide range of applications and services such as tracking, navigation, healthcare, emergency, etc. [1,2,3]. For instance, an improved localization accuracy, which reduces emergency response time by one minute, saves over 10,000 lives annually in the USA alone [4]. Since people spend most of their time indoors, immense attention has been paid to indoor localization. Despite the fact that GPS is the standard localization system, it cannot be leveraged in indoor settings. This is due to the high levels of signal interference and reflection [5] that block the line of sight to the reference satellites. Therefore, indoor localization has been an active research topic to find a ubiquitous and accurate alternatives to GPS in indoor settings [6,7,8,9].

Various technologies have been investigated, including Wi-Fi, radio frequency identification (RFID), Bluetooth, ultrawideband (UWB), cellular, zigbee, IMU, etc. [10,11,12,13]. Each technology has its own advantages which support its adoption in specific types of applications. Wi-Fi has been widely adopted due to its ubiquitous coverage and the support of the IEEE 802.11 standard by the majority of mobile devices [8,14,15,16,17,18,19,20,21,22].

Various localization techniques have been proposed to overcome the challenges related to indoor localization. These techniques include multilateration, fingerprinting, angle of arrival, and time-based techniques [23,24]. Fingerprinting and time-based techniques are the most researched ones in the field. Fingerprinting [25,26,27] is widely adopted due to its relatively good performance especially with deep learning. Fingerprinting technique builds a fingerprint database that involve signatures of Wi-Fi signals collected at different reference locations covering the area of interest. The fingerprint database is then used to define a model that can be used to estimate the user location, given the received signals at the run-time. Specifically, the defined model can be classified as deterministic [3] or probabilistic [28], or even as a machine-learning-based model [29]. Probabilistic solutions in general have better ability to mitigate the inherent random noise of wireless signals compared to deterministic solutions [24]. However, probabilistic solutions usually assume access points (APs)-independency to avoid a dimensionality problem [30], which is practically incorrect and also leads to information loss [24]. Therefore, deep learning has been widely adopted to learn the underlying joint distribution of signals received from the installed APs, leading to a superior localization performance. While many researchers have also examined fingerprinting techniques as a solution for localization issues, these techniques usually suffer from RSSI fluctuations and signal interference due to their sensitivity to obstacles, multipath fading, and indoor radio noise and/or hardware.

To overcome the above fingerprinting challenges, time-based techniques have been investigated. These techniques determine the distance of a mobile unit (e.g., phone) to APs using the measurement of the signal’s propagation time and the known signal’s velocity. Different approaches have been proposed for measuring the propagation time, including time of arrival (ToA) [31,32], time difference of arrival (TDoA) [33], and RTT [34]. The problem with ToA and TDoA is that they require a precise time synchronization of all devices. In contrast, RTT uses one clock to measure the time needed for the signal to travel to a destination node and return, and thus the synchronization problem is mitigated. Unlike RSSI-based approaches, RTT is more resilient to the challenges of cluttered indoor environments, including multipath interference, signal attenuation, transmission power variation, and radio interference. The fine time measurement (FTM) protocol, which can measure the RTT between the mobile phone and the APs, has recently been introduced by the IEEE 802.11-2016 standard. This protocol has increasing support from commercial APs and consumers’ mobile phones, making time-based techniques a promising solution for enabling practical indoor localization.

Nevertheless, RTT does not eliminate the significant indoor localization errors due to multipath propagation and latency as well as non-line-of-sight transmissions. Thus, signals traverse longer indirect paths, resulting in longer travel distance estimation (distance overestimation) [17]. To mitigate this issue, some solutions were proposed based on map matching or filtering [34]. However, they only slightly improve the localization performance [17].

In this paper, we present *RRLoc*: a hybrid fingerprinting-based indoor localization system that combines the advantages of both RSSI-based and time-based techniques. Specifically, the system constructs a fingerprint map of both RSSI and RTT at different discrete reference points in the area of interest. Then, the fused fingerprints are utilized for training a deep-learning-based classifier to track the user’s location.

This approach enables *RRLoc* to overcome the shortcomings associated with each modality. Nevertheless, fusing the RSSI and RTT to obtain a discriminative signature that facilitates the location estimation process is challenging, since they represent different modalities that are intrinsically dissimilar in nature. Thus, the error of the modality joint combination usually confuses/deceives the localization model, leading to more errors than those of each individual modality [14]. Therefore, *RRLoc* proposed a novel fusion and feature extraction method that automatically projects the two modalities into hyperspace where their correlation is maximum. To obtain that space, *RRLoc* employs a deep version of canonical correlation analysis (DCCA) [35,36] that yields a coordinated representation of the input modalities. The DCCA is trained to maximize the correlation between different RSSI and RTT, leading to separable representations facilitating user location estimation. In this vein, the developed hybrid model can cope with the localization challenges such as NLOS (non-line of sight), interference, clock synchronization, and signal attenuation. Unlike data integration methods [14], which tend to leverage information from one modality to improve the other, the proposed fusion method incorporates both modalities in a combined analysis, thus allowing for true interaction between them that maximizes their benefit.

The proposed system leverages the power of deep learning [4,29] in learning complex mapping functions to boost the robustness of the localization system. Furthermore, we ensure the system’s generalization by training models on different types of noisy signals during the location estimation stage. Additionally, *RRLoc* is able to avert the overfitting that may occur during the training phase by incorporating several regularization techniques.

We implemented and evaluated *RRLoc* using different Android phones on two different cluttered environments, a large environment of an area of 629 m${}^{2}$ and a small one of 141 m${}^{2}$. Seven commercial Google Wi-Fi APs were installed in each environment that already included other traditional non-RTT-enabled APs that could act as an added source of interference. Our results show that *RRLoc* achieved a submeter localization accuracy for both indoor environments with a median localization error of 0.42 m and 0.32 m for the two environments, respectively. These results reveal an improvement over the traditional RSSI fingerprinting accuracy by at least 267% and outperform the ranging-based multilateration localization approach accuracy by at least 496%. This accuracy was maintained under heterogeneous devices which qualified *RRLoc* as a robust and accurate indoor localization technique.

The contribution of this paper is threefold. First, we present a novel deep-learning-based indoor localization system exploiting the availability of the FTM protocol on consumer devices. Second, a novel data-driven-based fusion and feature learning method is designed for extracting a correlated representation of RTT and RSSI. Third, we train a robust localization model to enable pinpointing the user in the continuous space. We experimentally evaluate the performance of the proposed system, demonstrating its capability to localize with fine-grained accuracy.

The rest of this paper is structured as follows. Section 2 discusses the most relevant work of the proposed system. Section 3 provides a brief introduction to the IEEE802.11-2016 FTM protocol and the canonical correlation analysis approach. In Section 4, we provide a general overview of the *RRLoc* system architecture and present its different components. In Section 5, the modules of the *RRLoc* are introduced in details. Section 6 evaluates the different parameters of the system and shows its overall performance compared to the other approaches. Finally, Section 7 concludes the paper and discusses future work.

## 2. Related Work

Towards constructing smart buildings, many researchers have considered the problem of indoor localization. GPS has been known as an excellent technology used for localization, but it is not suitable for indoor positioning because indoor environments have great loss in signal propagation. Thus, many other sensors and technologies have been examined. Examples include Bluetooth, ultrasound, RFID, etc. However, the use of these sensors and technologies is restricted due to their limited energy, high cost, and/or constrained bandwidth. On the other hand, Wi-Fi technology has been recently given considerable attention with the great and continuous use of smart phones in almost all life activities. Thus, many Wi-Fi-based systems can be easily developed with reasonable cost as no special infrastructure is needed. The RSSI and time-based techniques are among the most applicable Wi-Fi techniques. Some discussions about these techniques and their relevance to our work are introduced in this section.

### 2.1. RSSI-Based Techniques

RSSI-based localization works by estimating the distance between two nodes and measuring their received signal strength. The RSSI positioning techniques are burdened by their poor accuracy that is due to many reasons such as NLOS, fading, noise data, etc. [37,38]. The simplicity of RSSI techniques has motivated many researchers to work on mitigating these issues. The RSSI-fingerprinting technique is one of the successful techniques toward this goal [25,26,27]. Fingerprinting localization approaches are common examples of probabilistic localization techniques. The RSSI-fingerprinting systems work on determining object location using two stages: offline and online phases. In the offline phase, the fingerprints of objects (objects’ RSSI) are measured at predefined reference positions to build a fingerprint database. These fingerprints will be used in the online phase to estimate the objects’ positions at new locations. Different features can be implemented as system fingerprints. Correspondingly, channel state information (CSI) techniques work on providing detailed information about the signal information between two communicated nodes. Both the RSSI and CSI localization approaches are highly affected by the changes in the power of Wi-Fi nodes which are very common. The heterogeneity of the used Wi-Fi devices may also degrade their performance. Although fingerprinting approaches are extensively used in developing good indoor localization systems overcoming the abovementioned challenges, the associated fingerprints/signatures are sensitive to signal interference, diffraction, and fading. In addition, to achieve efficient localization with fingerprinting, a homogeneous distribution of APs is highly recommended.

### 2.2. Time-Based Techniques

Alternatively, the time-based approaches are popularly used for indoor localization. They work to determine the objects’ positions based on time measurements and the known velocity of the transmitted signal. The ToA [31,32], TDoA [33], and RTT [34] are among the most popular techniques used for this purpose.The ToA technique works by measuring the time the signal takes to reach the receiver station (timestamp). In order to obtain accurate time estimation, a strict synchronization between the two sides is necessary [31,32]. The TDoA techniques work by transmitting signals from three or more stations and then measuring the difference between the signals propagation times, which are then used to estimate the user location. Again, this requires a type of time synchronization but with transmitters only, unlike the ToA technique [33]. The ToA, and TDoA techniques are considered one-way measurement techniques.

On the other hand, RTT is a two-way measurement technique that works by measuring the round-trip time, which is the time the signal takes to travel from the transmitter to the receiver and back. The most important advantage of the RTT technique over the ToA and TDoA techniques is that it does not require synchronization between the transmitter and receiver as only one clock is used. However, traditional multilateration systems that incorporate the RTT measurements suffer from poor accuracy due to NLOS and multipath effects [34].

Numerous Wi-Fi-RTT approaches have been developed to mitigate the effect of NLOS and multipath effects. In [39] for example, a real-time ranging Wi-Fi-RTT model was developed to reduce the error caused by multipath, and NLOS effects. In addition, in [40], the authors proposed a calibration model that works by eliminating the transmitter RTT range offset and thus improving accuracy. In [41], a Wi-Fi FTM geomagnetic positioning approach was proposed to mitigate the effect of NLOS. An enhanced mind evolutionary algorithm (EMEA) was incorporated in the developed approach to ensure the localization accuracy, whereas in [42], a Wi-Fi-RTT-based approach was developed by line-of-sight identification and range calibration. Some other works depend on identifying the NLOS and multipath signals and categorizing them to low- and high-quality signals using support vector machines [43].

For the sake of obtaining better positioning accuracy, a RTT-RSSI technique was proposed in [9]. This hybrid technique uses a new and simple multilateration model that combines RTT and RSSI techniques to improve the localization accuracy. However, this cannot be continually achieved since some signal attenuation may occur due to NLOS conditions. In addition, in [14], a hybrid RTT-RSSI fingerprinting localization approach was proposed. However, the deduced results show that the proposed approach is not able to achieve the expected accuracy as the correlation between the different modalities is not taken into consideration.

In this paper, a novel hybrid RTT-RSSI fingerprinting approach is presented. The introduced model is able to cope with the localization challenges mentioned above. The deep learning is incorporated in the designed model to increase its robustness and generalization. The details of the presented approach are given below.

## 3. Background

### 3.1. Round-Trip Time (RTT)

The round-trip time is a time-based technique mainly used for calculating distance. It is used in the current study to measure the distance between two Wi-Fi stations: in our case, the user’s mobile device and AP. The great advantage of using the RTT technique is its ability to measure the distance between two stations without necessitating explicit synchronization. It is worth noting that synchronization is one of the most important challenges of time-based localization. The RTT technique has been supported recently by the development of the FTM protocol in IEEE 802.11-2016.

The user’s mobile device (the initiator) starts the process by sending a Wi-Fi signal to the AP (the receiver) to check its availability. The receiver confirms its availability by sending an acknowledge signal. A two-way communication is thus started between the two stations to measure the distance. This communication can be repeated several times for the sake of obtaining a more accurate distance estimation. The RTT distance estimation is performed for all APs lying in the range of the mobile device. Another feature of using the RTT technique is its ability to compute the distance at the edge side and thus the user’s privacy is well preserved.

**Figure 1:**
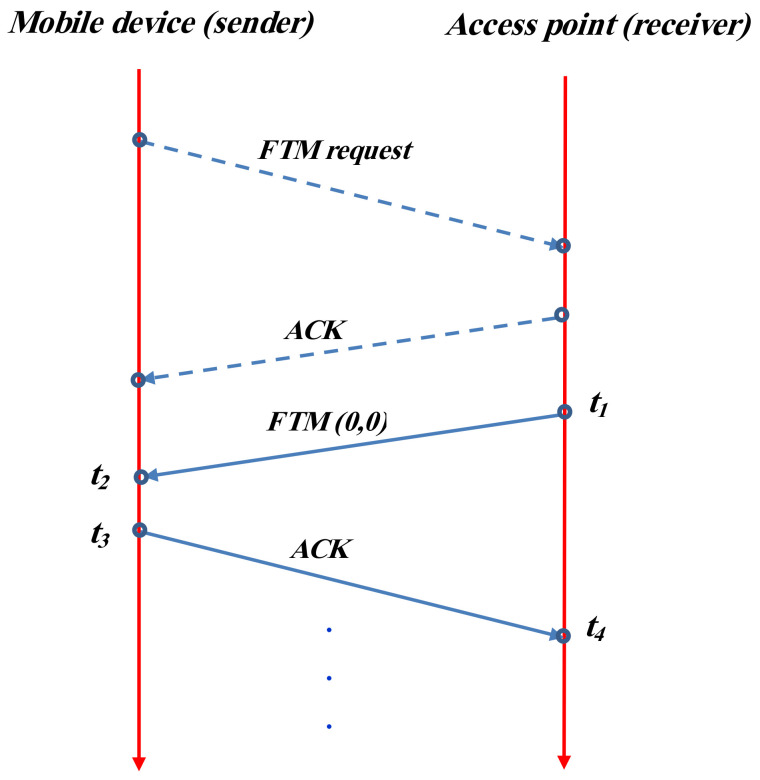
FTM protocol.

As shown in Figure 1, the process starts by sending a FTM request from the mobile device to the access point to see if it is available or not. The access point replies by the ACK signal if it is available and then the mobile device can compute the round-trip time by sending multiple FTM packets. The processing time at the mobile device side can be computed as follows:

(1) $$T_{p}=t_{3}-t_{2}\;$$

The round-trip time (RTT) can be computed as:

(2) $$\mathrm{RTT}=t_{4}-t_{1}-T_{p}$$

The distance (D) between the mobile device and the access point can be computed as:

(3) $$D=\frac{1}{2}\mathrm{RTT}\times C$$

where *C* is light speed which equals $3\times 10^{5}$ km/s.

It is noteworthy that the mobile device performs RTT ranging to all RTT-capable APs in the vicinity. Different from multilateration approaches [17,44,45], *RRLoc* harnesses the collected RTT values (via the FTM protocol) as fingerprints (signatures for each location) as described in the following section.

### 3.2. Canonical Correlation Analysis

In this section, we provide a brief background on the traditional canonical correlation analysis (CCA) on which the DCCA is built. The details of our DCCA algorithm are given in Section 5.

CCA [35,36] is a standard highly versatile statistical method for finding common correlation for two multivariate sets of variables (vectors) having the same situations. In particular, CCA **linearly** projects the input sets into another lower-dimensional space in which these sets are maximally correlated. This helps in studying the strength of the relationship between two quantitative variables and how they are related. An appealing property of CCA for prediction tasks is that if there is noise in either set, the learned representations should not contain that noise in the new space.

More formally, assume $S=\left[s_{1},s_{2},\ldots ,s_{N}\right]\in R^{d_{x}\times N}$ and $Y=\left[r_{1},r_{2},\ldots ,r_{N}\right]\in R^{d_{y}\times N}$ are two different multivariate variable sets of *N* samples and feature space of dimensions $d_{x}$ and $d_{y}$, respectively. The goal of CCA is to find *K* pairs of linear projections (canonical vectors) $W_{s}=\left[w_{s,1},w_{s,2},\ldots ,w_{s,K}\right]\in R^{d_{x}\times K}$ and $W_{r}=\left[w_{r,1},w_{r,2},\ldots ,w_{r,K}\right]\in R^{d_{y}\times K}$, so that the correlations between $W_{s}^{T}S$ and $W_{r}^{T}R$ are maximized. Specifically, CCA aims at finding the projection matrix that maximizes the correlation coefficient α between $W_{s}^{T}S$ and $W_{r}^{T}R$ as:

(4) $$\alpha \left(W_{s}^{T}S,W_{r}^{T}R\right)=\frac{W_{s}^{T}SR^{T}W_{r}}{W_{s}^{T}SS^{T}W_{s}W_{r}^{T}RR^{T}W_{r}}$$

That is, we want to find:

(5) $$\left(W_{1}^{*},W_{2}^{*}\right)=\underset{W_{s},W_{r}}{\mathrm{argmax}}\frac{W_{s}^{T}SR^{T}W_{r}}{W_{s}^{T}SS^{T}W_{s}W_{r}^{T}RR^{T}W_{r}}$$

Since α is scaling-invariant, we can rewrite the correlation as:

(6) $$\left(W_{1}^{*},W_{2}^{*}\right)=\underset{W_{s},W_{r}}{\mathrm{argmax}}W_{s}^{T}SR^{T}W_{r}$$

$$s.t.\;W_{s}^{T}SS^{T}W_{s}=1,\;W_{r}^{T}RR^{T}W_{r}=1$$

To find the optimum solution for Equation (6), one has to solve the general eigenvalue problem of the form [46]:

(7) $$\left[\begin{array}{cc} 0 & \Sigma_{sr}\\ \Sigma_{rs} & 0 \end{array}\right]\left[\begin{array}{c} W_{s}\\ W_{r} \end{array}\right]=\lambda \left[\begin{array}{cc} {\hat{\Sigma }}_{ss} & 0\\ 0 & {\hat{\Sigma }}_{rr} \end{array}\right]\left[\begin{array}{c} W_{s}\\ W_{r} \end{array}\right]$$

where ${\hat{\Sigma }}_{ss},{\hat{\Sigma }}_{rr}$ are the covariance matrices. $\Sigma_{sr}$ and $\Sigma_{rs}$ are defined as: $\Sigma_{sr}=\frac{1}{N}SR^{T}$ and $\Sigma_{rs}=\frac{1}{N}RS^{T}$.

By solving Equation (7), we obtain *K* eigenvectors ${\left\{\left[W_{s,k};W_{r,k}\right]\right\}}_{k=1}^{K}$ and the corresponding Kth eigenvalue that is equal to the correlation coefficient in Equation (4). Therefore, the aimed projection matrix *W* is the set of obtained eigenvectors.

In this paper, we adopt a deep-learning-based version of CCA, denoted DCCA [47], that can be viewed as a **nonlinear extension** of the traditional CCA.

## 4. System Overview

**Figure 2:**
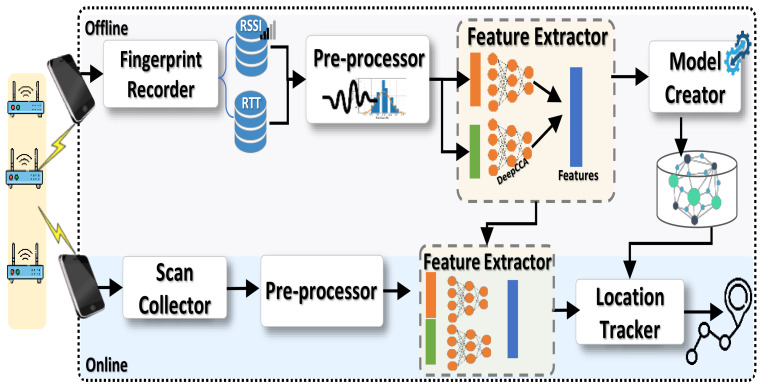
*RRLoc* system architecture.

Figure 2 shows the *RRLoc* system architecture. *RRLoc* works in two stages: an offline calibration and training stage and an online localization stage. During the calibration stage, Wi-Fi data is collected at predefined reference points that uniformly cover the area of interest (This data can be transparently collected without the burden of site surveying using our earlier work in [48]). Typically, the collected data constitutes the fingerprints of each reference point involving the RTT and RSSI measurements from the overheard APs. This fingerprint map is constructed using the **Fingerprint Recorder** App running on a mobile phone and leverages the Android RTT API [49] to scan for RTT and RSSI readings. The collected fingerprint map is uploaded to an online running service for further processing. The **preprocessor** module is used to construct pairs of fixed size vectors (i.e., RSSI and RTT vectors) that are simultaneously captured from the APs overheard in the area of interest. Each pair of vectors is then forwarded to the **feature extractor** module to extract high-level location discriminative features. Specifically, this module learns the complex nonlinear transformation of the original low-level features to a new feature space where the RSSI and RTT projections are highly correlated as described in Section 5.2. Then, the obtained features are fed to the **localization model creator** module that is responsible for training a localization model for estimating the location of the mobile device. The output of this calibration stage is two trained models (i.e., the deep canonical correlation analysis model and the localization model) that are stored for later retrieval in the online stage.

During the online phase, users are tracked in real-time. When carrying their mobile phones at unknown locations, the phones scan for the APs in the vicinity. Each scan includes the RSSI and RTT from the overheard APs which are forwarded to the *RRLoc* server. This data is first handled by the **preprocessor** module to form the pairs of unified length vectors. Thereafter, these pairs are fed to the trained **DCCA model** to extract the desired features. Finally, the **location estimation model** feeds the extracted features to the localization model trained in the calibration stage to estimate the most probable reference locations where the user may be located. Based on these probabilities, the system obtains the user’s location in the continuous spatial space.

## 5. The *RRLoc* System

Figure 2 shows the different modules of the *RRLoc* system. In the balance of this section, we describe the details of each module. Table 1 summarizes the notations used in this section.

### 5.1. The Preprocessor Module

The preprocessor module is responsible for mapping the RTT and RSSI measurements to a pair of fixed-length feature vectors. Each entry in the feature vector represents a measurement from an AP such that an AP entity in the RTT vector has a corresponding value in the RSSI vector. It is worth noting that all the installed APs cannot be overheard at every scan due to the range. Thus, only a subset of the APs may be detected in an arbitrary scan leading to variable-length feature vectors. To resolve this issue, nonheard APs in a specific scan are substituted by the RTT value of $0.2\times 10^{-3}$ ms, which is equivalent to a 60 m distance. This value is larger than any RTT value for the APs in the scanning range. Similarly, the RSSI value of −100 dBm is assigned to any unheard AP as it is lower than all RSSI values received from within the range of APs in the collected scans. Thus, a short RTT/low RSSI value is assigned to any AP lying far away from the mobile device carried by the user. It is also observed that when the mobile device is very close to an arbitrary AP, a negative distance is reported by the Android API [49]. This can be explained due to the internal configuration and calibration of the Wi-Fi cards or the multipath compensation algorithms that process the measurements in firmware before the driver receives them. RTT may also suffer from some latency when used with fast moving mobile devices. The presence of such negative values (former case) or latency (latter case) usually leads to a significant drop in the performance of traditional multilateration approaches [17]. However, this event cannot affect the *RRLoc*’s performance as it is a fingerprinting-based technique, and such negative values or delay can be considered a strong signature of particular locations.

Finally, normalization is employed to rescale the input values of each modality to be in the range between [0, 1]. Normalization has been empirically verified to speed up model convergence during training [50].

### 5.2. The Feature Extractor Module

This module aims to transform the preprocessed RSSI feature vectors and the corresponding RTT vectors to a latent space in which they are highly correlated. This is a more flexible feature-based fusion approach of different modalities given their spatial dependency while avoiding spurious measurements. In other words, both the RTT and RSSI of an arbitrary AP are different representations of how far the mobile device is from that AP. Therefore, projecting the two modalities into another hyperspace where intercorrelation is maximum leads to more separable location signatures and thus better localization.

To discover that latent space, we adopt DCCA [47]. Traditional techniques, e.g., [14] obtain a joint multimodal representation by concatenating the individual RSSI and RTT vectors. Despite the simplicity of that approach, it loses essential information about the correlation (i.e., spatial dependency) between the input modalities. Moreover, compared to a single modality, the joint representation usually leads to a worse performance due to the presence of different types of noise and latency associated with data of varying nature. Compared to the classical CCA [35,36], which linearly transforms the input views into highly correlated projections, the DCCA solves the same objective function by realizing more powerful **nonlinear** projections in a new latent space using deep neural networks. These projections are learned via the gradient descent technique. The intuition behind leveraging the deep version of CCA is the ability of the deep neural network to learn complex relations from such noisy Wi-Fi data automatically. Unlike traditional deep-learning methods that are trained to maximize the likelihood of target class (location) given the RSSI scan alone [29] or the RTT scans alone [34], *RRLoc* combines both modalities using the correlation-based objective function of the DCCA. This empowers the **system robustness and learning ability** compared to just concatenating the noisy raw measurements that may deceive the localization model (as evaluated in Section 6.2.3).

**Figure 3:**
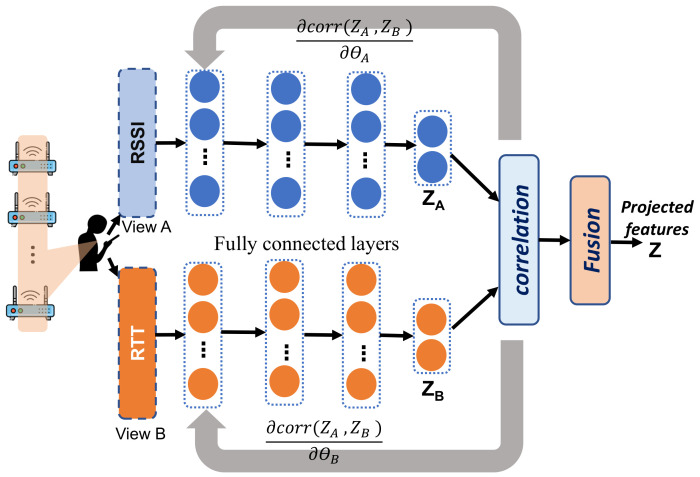
The network structure of the DCCA-based feature extraction module. It consists of two deep networks learned so that the output layers (topmost layer of each network) are maximally correlated. A correlation layer is stacked on top of a fully connected layer to calculate the correlation between the views.

Figure 3 shows the schematic structure of the proposed DCCA feature extraction model. As shown in the figure, the DCCA consists of two independent deep neural networks (DNNs), one for each type of measurements (RSSI and RTT). Each DNN consists of cascaded fully connected layers. The input layer of the DNN *A* and DNN *B* are the RSSI and RTT vectors which are captured simultaneously by the mobile device. These DNNs are then trained to encode these inputs to a fixed-size subspace where the corresponding output vectors ($z_{A}$ and $z_{B}$) are maximally correlated. Specifically, let $S_{A}$ be a set of RSSI input vectors, $S_{B}$ is the corresponding set of RTT vectors which are collected simultaneously at the same set of reference points. These modalities are fed to the DCCA twin networks to obtain the aimed latent representations that leverage the advantages of both modalities. For instance, the output of the first layer of network *A* is $h_{1}^{A}=\sigma \left(W_{1}^{A}S+b_{1}^{A}\right)$, where σ is a nonlinear activation function (e.g., logistic Sigmoid) applied component-wise, $W_{1}^{A}$ is a matrix of weights and $b_{1}^{A}$ is a vector of biases. The output of each layer is used to calculate the output of the next layer until the final layer *d* whose output is calculated based on the output of the previous layer $h_{d-1}$ as $f_{A}\left(S_{A}\right)=\sigma \left(W_{d}^{A}h_{d-1}+b_{d}^{A}\right)$ which is the intended latent representation (*z*), i.e., the spatially correlated feature vector. Similarly, the representation obtained by the second DNN of *g* layers is $f_{B}\left(S_{B}\right)=\sigma \left(W_{g}^{B}h_{g-1}+b_{g}^{B}\right)$ with different parameters $W_{g}^{B}$, $b_{g}^{B}$ and *g*. The objective of the DCCA is to jointly learn the parameters $\theta_{A}$ and $\theta_{B}$ for both neural networks such that the correlation between $z_{A}$ and $z_{B}$ is maximum. Therefore, the objective function of the DCCA is defined as follows:

(8) $$\left(\theta_{A}^{*},\theta_{B}^{*}\right)=\underset{\left(\theta_{A},\theta_{B}\right)}{\mathrm{argmax}}Corr\left(f_{A}\left(S_{A};\theta_{A}\right),f_{B}\left(S_{B};\theta_{B}\right)\right)$$

To achieve this, we compute the correlation and its gradient with respect to the output layers. Then the back-propagation is used to update the parameters of both networks. This process is repeated until convergence is obtained (Given that, in general, optimization of deep models may not achieve the best performance if the model parameters are initialized randomly. Therefore, we adopt the Xavier initialization approach in [51] for better initialization of *the feature extraction* module).

After the training of the twin networks, the transformed feature vectors $z_{A}$ and $z_{B}$ become in the coordinated hyperspace. Then, these vectors are fed to the localization model as input for weighting with respect to their contribution in the location recognition process (as discussed in Section 5.3).

### 5.3. Location Estimation Module

This module is responsible for utilizing the correlation features (*z*) extracted from the DCCA network to train the localization model and find its optimal parameters. The trained model is used in the online phase by the *online location predictor* module.

**Figure 4:**
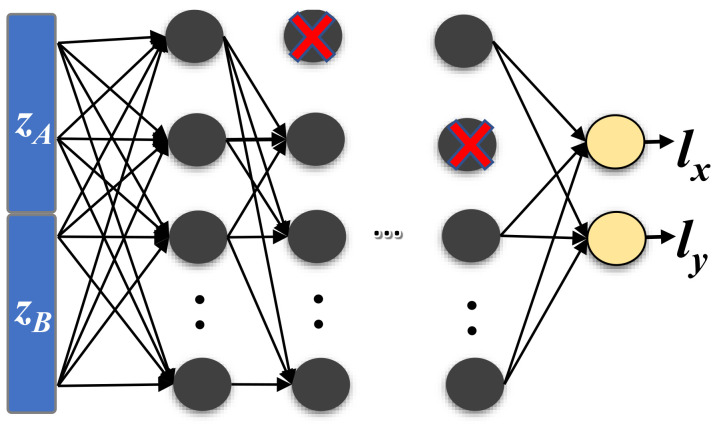
The network structure of the location estimator module. Crossed neurons represent dropped out units to avoid overfitting during training.

Figure 4 shows the structure of the considered deep neural network for localization. Specifically, *RRLoc* adopts a fully connected feed forward neural network. The hierarchical representation of *RRLoc* is obtained by cascaded hidden layers of nonlinear processing units. The rectified linear unit (ReLU) (the state of the art of nonlinearity) is used as the activation/transfer function for the hidden layers due to its sparsity and immunity to vanishing gradient problems [52].

The input layer of the network is a vector *z* of length *v* which is obtained from the *feature extraction* module (described in Section 5.2). The network is trained to operate as a regression model having an output layer consisting of two neurons (corresponding to the 2D spatial coordinates ($l_{x},l_{y}$). Therefore, the selected network can be classified as a many-to-one, i.e. the model will learn a function that maps the latent representation *z* of the RSSI and RTT to an output location.

One advantage of designing the *localization model* to operate as a regressor rather than a classifier is the requirement to estimate the user location in the continuous space. Classification models can only estimate the user locations at one of the predefined few discrete reference points. This usually leads to a bad user experience as the predicted locations will be spaced out even with a very accurate model. To ensure the required smooth tracking of the users in the continuous spatial space (*RRLoc* can locate the user anywhere, even on locations different from reference points.), *RRLoc* models the localization process as a deep-learning-based regressor that is trained to estimate the user location coordinates in the environment (even the nonsurveyed ones). We utilized the Adam optimizer [53] and mean square error (MSE) as a loss function.

DNN are known to have a tendency to overfit the training data, reducing their predictive skill [54]. Therefore, we utilize two regularization techniques: First, we use *dropout* to probabilistically exclude neurons and their connections from activation and weight updates while training a network. Second, we leverage *early stopping* so that training would terminate once the validation set no longer obtains performance improvements [55].

### 5.4. Online Phase

The goal of this phase is to track the users’ locations in the environment of interest. Initially, each user device captures the RSSI and RTT from the detectable APs in the environment, and forward the scan to our running service to preprocess and extract the coordinated feature vectors from the trained DCCA, as described in Section 5.2. These vectors are then fed to the trained localization model to obtain a location estimate in the continuous space.

## 6. Evaluation

In this section, the data collection setup and tools used are described first. Then, we show how the system performs by varying the different system parameters. Finally, we compare the performance of *RRLoc* to the state-of-the-art techniques.

### 6.1. Collection Setup and Tools

**Figure 5:**
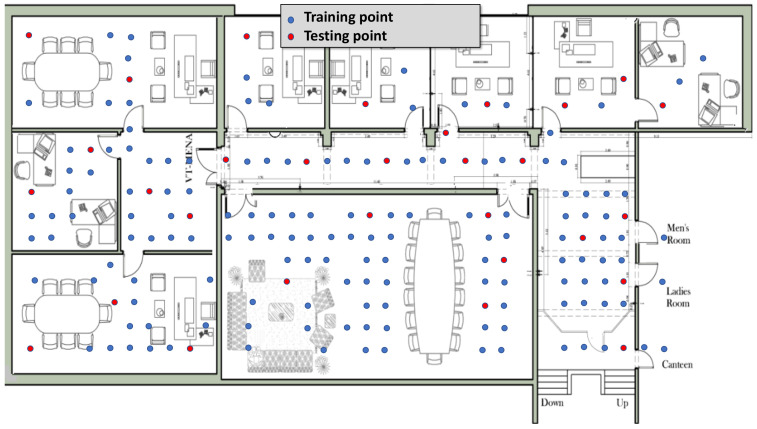
Layout of Lab testbed. Blue and red circles represent training and testing points, respectively.

**Figure 6:**
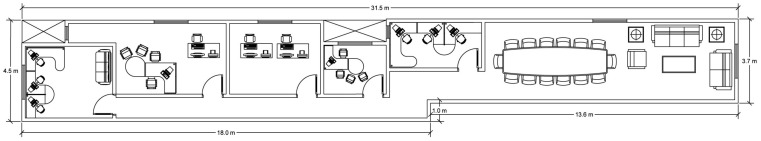
Layout of the Office testbed.

For analyzing and evaluating the *RRLoc* system performance, we deployed the system in two realistic indoor testbeds. Table 2 summarizes the characteristics of the two testbeds. The first one, denoted as “Lab”, is a full floor in our university campus which spans an area of 629 m${}^{2}$ and contains nine rooms of different sizes and a long corridor as shown in Figure 5. The second testbed, denoted “Office”, as shown in Figure 6 is an administrative building of 141 m${}^{2}$ area consisting of a large meeting room, a long corridor, and five rooms. We used a wireless network setup of seven Google Wi-Fi APs uniformly distributed to cover the whole area of interest in both testbeds. The area of interest in both testbeds was uniformly discretized into different reference points distributed over the area one meter apart from each other. (We evaluate the effect of changing the spacing between reference points later in this section.) The Lab testbed had 143 different reference locations, while the second testbed included 76 locations. Each reference location was ensured to be covered by at least one Google Wi-Fi AP.

Data were collected with an Android application installed on different Android phones including Google Pixel XL and a Pixel 2XL. The application continuously scanned for the nearby APs. To facilitate ground-truth profiling, our data collector application ran synchronously on all mobile devices with one device dedicated to controlling ground-truth collection for all devices. The user input the coordinates of his current location (ground-truth) and launched the data collection process. At each reference location, at least 100 samples were captured in 3 min for training purposes. Hold-out test sets were collected independently, including 21 and 30 locations (different from the training points) in the Office testbed and the Lab testbed, respectively. This was completed over several days during working hours (to consider the time variation of signals indoors).

### 6.2. Effect of Changing RRLoc Parameters

In this section, we study the impact of the different system parameters including the deep model’s hyperparameters on the *RRLoc* performance and how much they enable learning the nonlinear transformations for achieving the maximum correlation between all modalities and thus better localization accuracy. These parameters include the number of layers, the effect of the feature extraction method, and the size of the feature vector. In the following subsections, we show the effect of varying these parameters only on the Lab testbed for clarity of presentation. However, we present how *RRLoc* performs in both testbeds in SubSection 6.3.1. Table 3 summarizes the default values of system parameters that are used throughout the evaluation section.

#### 6.2.1. Number of Layers in the Network

**Figure 7:**
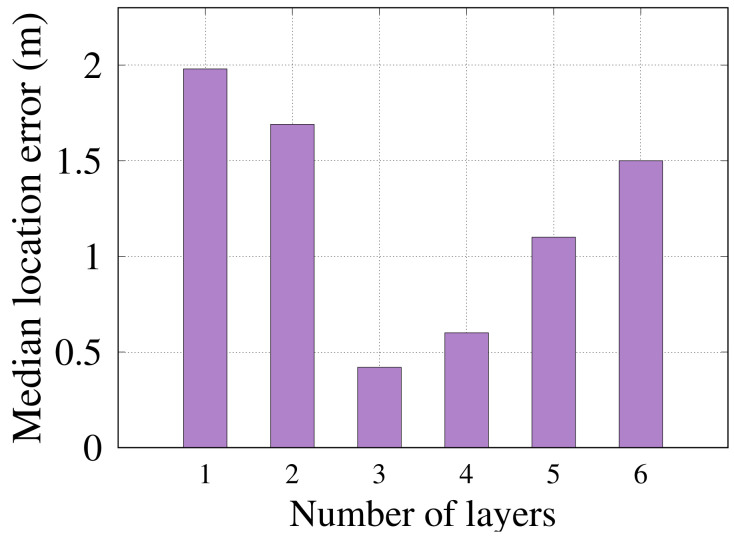
Effect of changing the number of layers on *RRLoc* localization error.

Figure 7 shows the effect of changing the number of layers on *RRLoc* performance. As shown, the more hidden layers to consider, the better the accuracy (i.e., less localization error) *RRLoc* can achieve until it reaches an optimal value at three layers. This can be justified due to two reasons. First, increasing the number of layers increases the distributed learning ability of the localization model. In this vein, the model has enhanced computing power enabling the better fitting of the underlying function (without underfitting). Second, as few as three layers are enough to allow the localization model to learn the user location from the latent extracted features. It is worth noting that the extracted features obtained by the DCCA radically simplify the classification problem. Beyond three layers, the model tends to overfit the training data, reducing its flexibility and thus its accuracy. As a result, *three* layers is set as the default number of layers in the *RRLoc* model to achieve a balance between underfitting and overfitting.

#### 6.2.2. Dropout Percentage

**Figure 8:**
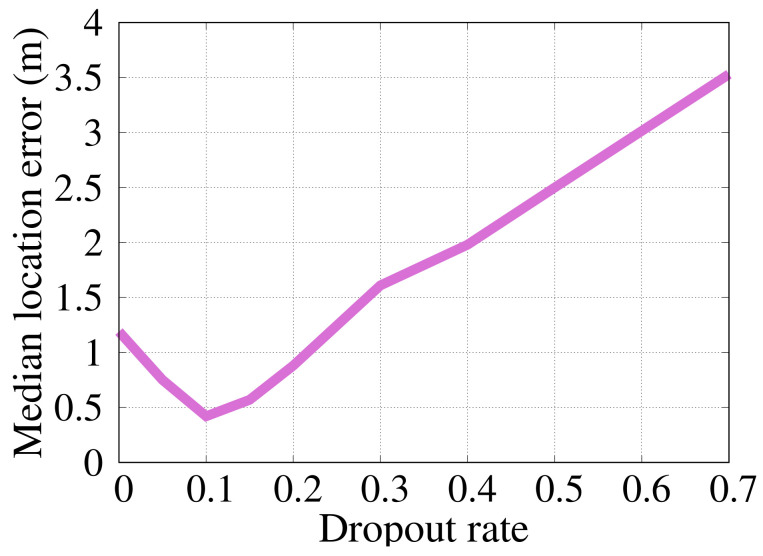
Effect of changing the dropout rate on *RRLoc* localization error.

The effect of increasing the percentage of dropout is shown in Figure 8. It can be observed from the figure that at a rate of 0.1 dropout, the best performance of *RRLoc* is achieved (even better than the case of no dropout). This confirms the significant regularization role of dropout in boosting the network learning to generalize rather than overfit the training data.

#### 6.2.3. Feature Extraction Method

**Figure 9:**
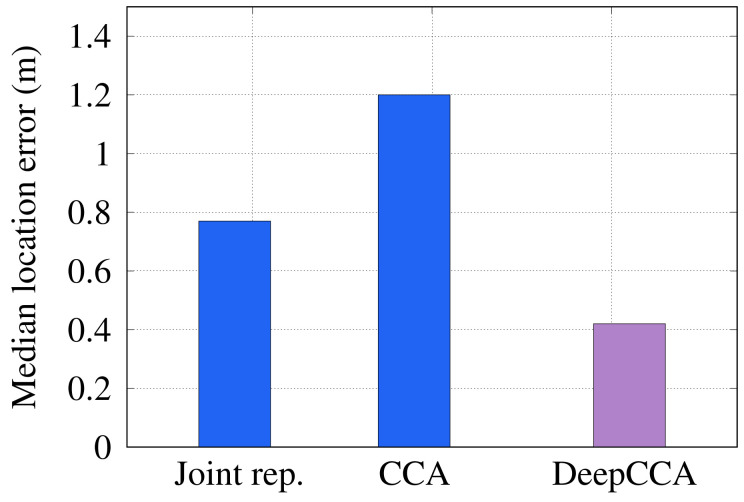
Effect of feature extraction module on *RRLoc* performance.

In this section, we study the influence of the different feature extraction techniques on the overall system performance. Figure 9 compares the effect of using DCCA for extracting discriminative features to either using joint representation (i.e., concatenated RSSI and RTT features) or feature projection using the classic CCA [36]. The figure confirms the favorable performance of the DCCA of *RRLoc* as compared to both the joint representation and classic CCA. Specifically, *RRLoc* gives an improvement of 83%, and 186% in estimating the correct user location as compared to the joint representation and classic CCA, respectively. This can be explained by noting that the classifier tries to learn the underlying distribution of the input and map it to the output. However, in the case of the joint representation, the classifier is supplied with mixed input of two different distributions, which generally yields an information loss of the correlation between the two modalities. On the other hand, the classical CCA assumes that a linear transformation of the inputs improves correlation between RTT and RSSI in the new space, which is not always the case in practice due to the propagation challenges in indoor environments. These results highlight the efficacy of using the DCCA in capturing the nonlinear correlated signatures of the two Wi-Fi input modalities, facilitating the accurate tracking of the user location.

#### 6.2.4. Feature Vector Length

**Figure 10:**
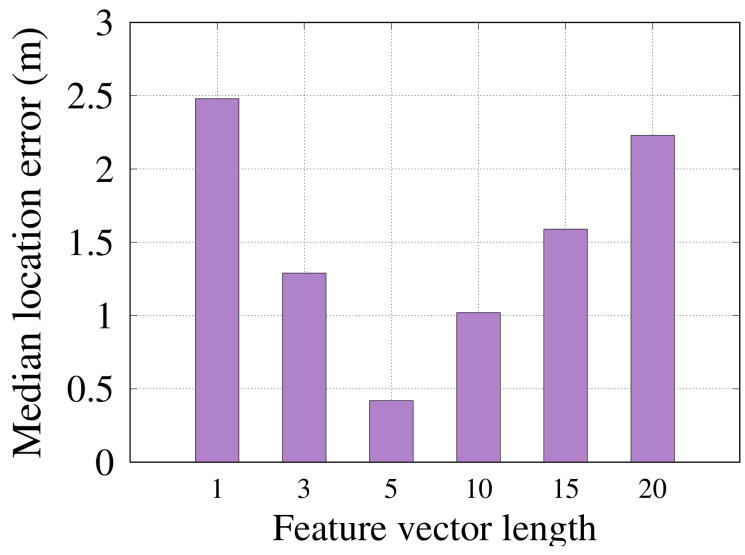
Effect of changing the feature vector length on *RRLoc* accuracy.

Figure 10 shows the location estimation accuracy of *RRLoc* as a function of the latent space dimension size obtained by the DCCA network. It is clear from the figure that increasing the size (dimensions) of the latent feature vector *z* improves the *RRLoc* performance. The figure also shows that a feature vector *z* of five dimensions yields the best performance. Beyond five dimensions, a performance deterioration is observed. This can be explained as the additional dimensions usually include undesired artifacts that reduce the correlation between the input modalities.

#### 6.2.5. Impact of Each Modality

**Figure 11:**
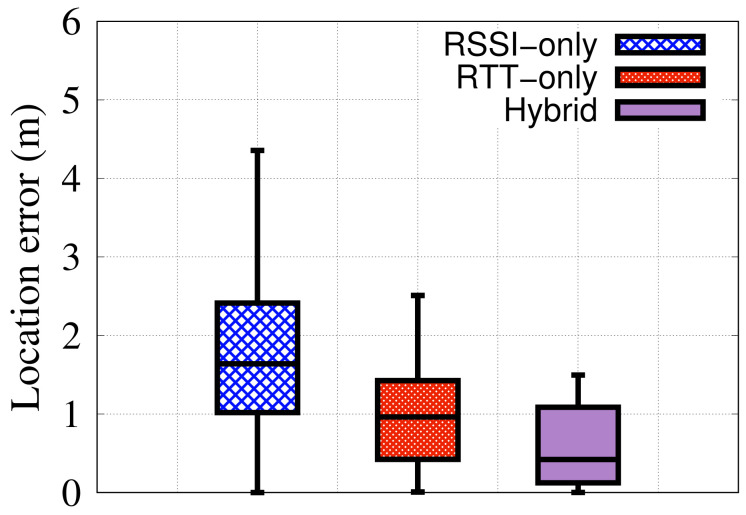
Effect of varying the considered modality on *RRLoc* performance.

Figure 11 shows the performance of the *RRLoc* system when an individual modality is used as well as the hybrid RSSI-RTT version. The figure shows the favorable performance of RTT compared to the RSSI. This can be justified by noting that the RSSI readings are more noisy compared to RTT due to its higher sensitivity to multipath effect. The combination of RSSI and RTT yields an improvement in the median accuracy by 289% and 129% compared to RSSI-only and RTT-only, respectively. This is due to leveraging the advantages of both modalities leading to a remarkable enhancement of the *RRLoc* accuracy in all percentiles. Specifically, the RSSI measurements boost the *RRLoc* performance compared to using the RTT counterpart alone in cases of the absence of a direct line-of-sight transmission, while RTT maintains the system robustness in case of noisy and fluctuating RSSI signals. These results validate the gain of such combination on the system performance.

#### 6.2.6. Effect of Access Points Density

**Figure 12:**
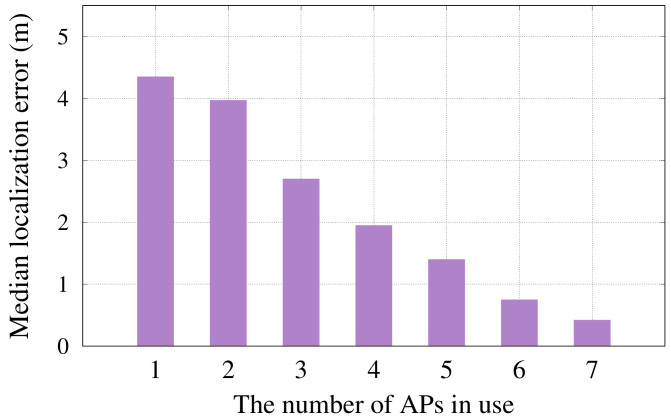
Effect of changing the number of access points on *RRLoc* performance.

Figure 12 shows the impact of varying the number of access points in use on the median localization error and response time (i.e., average time per location estimate). This experiment is performed by removing APs from the feature vector randomly. The figure shows that increasing the APs installed in the area of interest yields a better localization accuracy. This can be explained by noting that increasing the number of APs leads to richer feature vectors to accommodate the blocking that might occur to any connection between the transmitter and any receiver at the run time.

#### 6.2.7. Effect of Fingerprint Points Density

**Figure 13:**
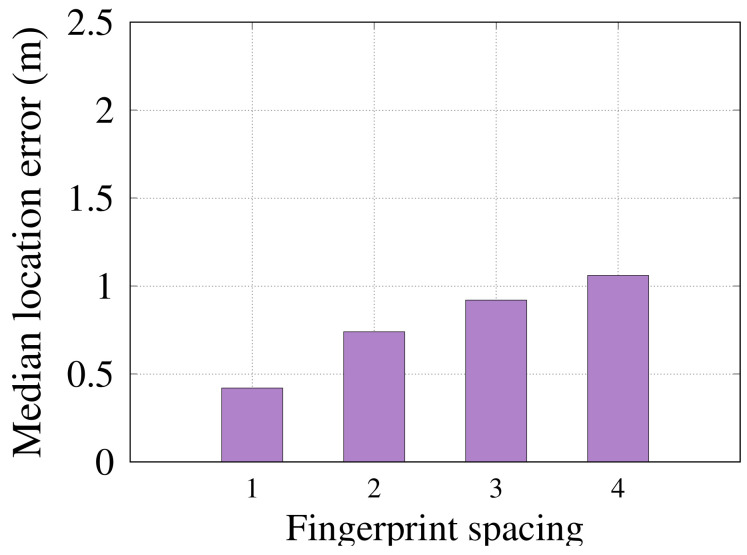
Effect of varying the spacing between reference points on *RRLoc* performance.

In this section, we study the effect of the density of fingerprint points in the area of interest on the *RRLoc* performance. Figure 13 shows that increasing the density (shorter spacing between points) of the fingerprint points leads to better localization accuracy. However, unlike traditional RSSI-based systems [29,48,56], increasing the spacing between fingerprint points leads to a slight decrease in the performance of *RRLoc*. In particular, the *RRLoc* localization accuracy loses just 32 cm by doubling the fingerprint spacing from 1 m to 2 m. This confirms the *RRLoc*’s ability to operate at lower fingerprint densities, leading to a drastic saving of time and effort associated with the data collection process.

### 6.3. Comparative Evaluation

In this section, the performance of *RRLoc* is compared to three Wi-Fi-based localization systems: WiNar [14], WiDeep [29] and ranging-based system [17]. WiNar [14] leverages a deterministic approach that matches the captured RTT measurements to the prerecorded fingerprint map to estimate the user’s location. WiNar leverages the RSSI to weigh the estimated locations. On the other hand, WiDeep [29] builds a RSSI-based localization system using the deep denoising autoencoder neural network. The ranging-based system in [17] uses the multilateration approach based on the RTT for enabling indoor localization while detecting NLOS. All techniques have been evaluated on the same data for a fair comparison.

#### 6.3.1. Localization Accuracy

**Figure 14:**
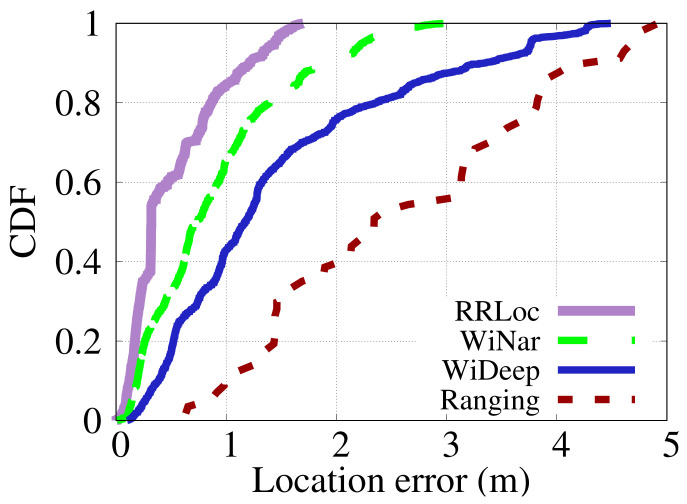
Comparison of CDFs of different systems in the Office testbed.

**Figure 15:**
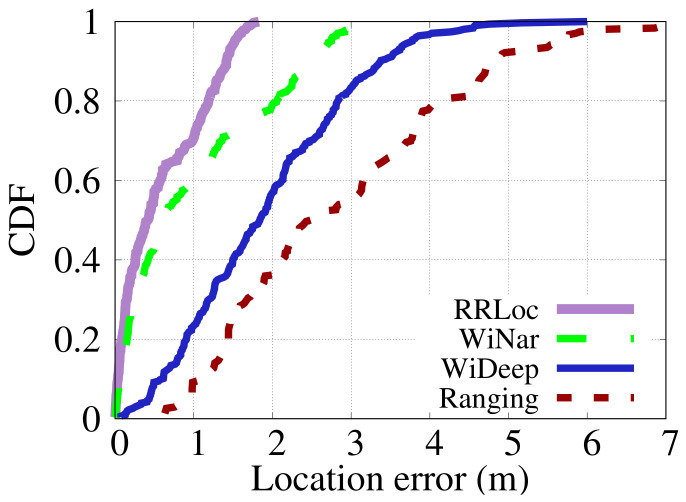
Comparison of CDFs of different systems in the Lab testbed.

Figure 14 and Figure 15 show the CDF of localization error of the different techniques in the two testbeds. Figure 14 shows that *RRLoc* gives an improvement in median error obtained in the Office testbed by 129%, 267%, and 632% compared to the WiNar [14], WiDeep [29] and ranging-based [17] systems, respectively. On the other hand, the performance of the different systems in the Lab testbed (i.e. the larger testbed) is shown in Figure 15. The results depicted in the figure show that *RRLoc* outperforms WiNar [14], WiDeep [29] and ranging-based [17] systems by 45%, 337% and 469%, respectively. In summary, as shown in Table 4 and Table 5, *RRLoc* improves all the percentiles upon the other system in both testbeds. This can be explained by noting that the concatenation-based approach of WiNar loses the correlation information between RSSI and RTT measurements. Moreover, the deterministic matching method adopted by WiNar cannot cope with the noisy measurements of both modalities. WiDeep leverages only RSSI measurements to train a powerful deep-learning model for localization purposes. Nevertheless, the accuracy of this approach depends on the quality of the captured signals, which are generally noisy in cluttered environments. The performance of a ranging-based multilateration system usually suffers from NLOS problems leading to coarse-grained accuracy. Different from these systems, *RRLoc* leverages the flexibility of the proposed DCCA-based approach to maximize the benefits of RSSI-RTT fusion. Additionally, *RRLoc* considers the spatial dependency between the two modalities through a powerful deep neural network that learns robust location-discriminative features given the inherent noise in each modality. This highlights the promise of *RRLoc* as the next generation of robust Wi-Fi-based positioning system.

#### 6.3.2. Time per Location Estimate

**Figure 16:**
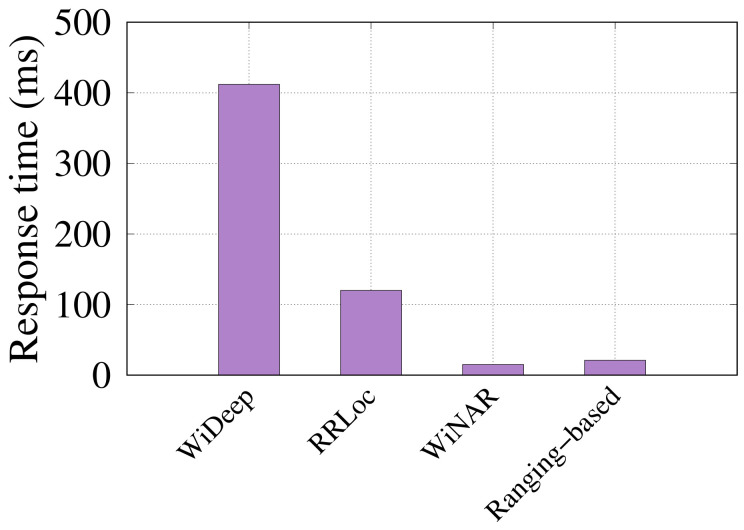
Comparison of run time of the different systems.

We used a a Lenovo Thinkpad X1 laptop running a 2.2 GHz Intel i7-8750H processor with 64 GB RAM, and a Nividia GTX1050Ti 4GB GPU for evaluating the running time of the different systems. Figure 16 shows the results. The figure shows that as *RRLoc* and WiDeep [29] are deep-learning-based systems, they need to pass the input through all the layers of the network to calculate a location estimate. This takes more time than the traditional deterministic method used in WiNar [14] and ranging-based [17]. On the other hand, *RRLoc* has running time that is remarkably less than WiDeep [29]. This is due to the fewer number of layers and neurons and, by extension, less calculations compared to WiDeep [29]. Nonetheless, all systems allow real-time tracking of the user which can be further enhanced (if needed) by parallelization.

#### 6.3.3. Device Heterogeneity

**Figure 17:**
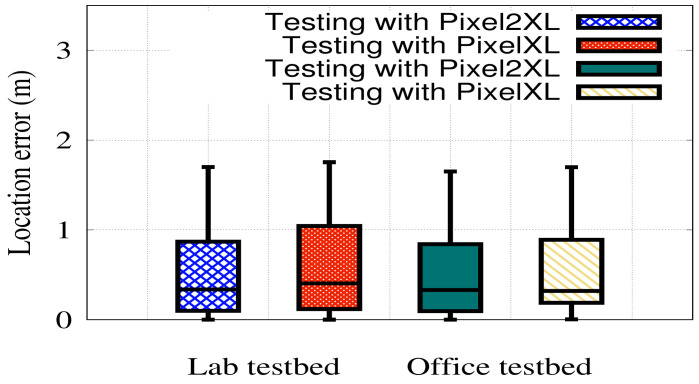
Effect of varying the testing device on *RRLoc* performance in the two testbeds.

In this section, we investigate the system robustness to device heterogeneity, where one device is dedicated to capturing training data, and the other is used for testing. Figure 17 shows the system performance when varying the testing devices in the two testbeds, i.e., in the case of testing with Pixel XL, the training device is Pixel 2XL and vice versa. The figure shows that *RRLoc* achieves a consistent performance in all cases for the two devices which is slightly better in the case of Pixel 2XL. This can be justified as different phones, in general, vary in hardware factors, e.g., form factors, chips, antenna locations, leading to a variation of the measured RSSI. The combined effect of these factors can be considered to be an offset that affects only the RSSI depending on the phone as addressed in [57,58,59]. However, hardware diversity has a negligible effect on RTT measurements [34]. The fusion of RSSI and RTT has shown to be effective in mitigating the effect of hardware diversity, leading to a more robust localization performance. It is worth mentioning that currently few devices support RTT scanning. However, the number of models of supported devices is increasing [49].

## 7. Conclusions

We presented *RRLoc*, a hybrid deep-learning-based indoor localization system which fuses fingerprinting and time-based techniques to combine the merits of both techniques. A novel approach is adopted for fusing RSSI and RTT measurements and extracting high-level features using deep canonical correlation analysis. The proposed RRLoc showed great capabilities in overcoming the challenges of these techniques in indoor environments even with the use of heterogeneous devices. Different modules are incorporated to improve the deep model’s generalization against overtraining and noise. The proposed system is evaluated in two different environments (office and lab). The RRLoc system achieved a submeter localization accuracy for both indoor environments with a median localization error of 0.42m and 0.32m respectively. RRLoc is able to improve upon all the percentiles of the other systems in both environments when using the coordinated representation of RTT and RSSI data. In the future, we plan to deploy *RRLoc* at scale and automate the data collection process to lessen the fingerprinting burden.

## Figures and Tables

**Table 1:** Notations used in the paper.

Notation	Description
*q*	The total number of access points covering the area of interest.
*m*	The number of access points detected in an arbitrary scan ≤q.
*n*	The total number of scans collected in the area of interest (i.e. at all locations).
*l*	The total number of reference location in the area of interest.
*s*	RSSI measurement vector which is composed of *q* entries, each of them represents the RSSI corresponding to one AP.
*r*	RTT measurement vector which is composed of *q* entries, each of them represents the RTT corresponding to one AP.
*w*	Matrix of weights.
*b*	Vector of biases.
*z*	The extracted feature vector as generated from the DCCA.
θ	The model parameters.
*v*	The size of the extracted feature vector.

**Table 2:** Summary of the testbeds considered in evaluating *RRLoc*.

Criteria	Lab Testbed	Office Testbed
Area (m${}^{2}$)	17 × 37	4.5 × 31.5
Number of training points	143	76
Number of testing points	30	21
Spacing of seed points (m)	1	1
Building Material	Brick	Brick& Wood
Number of APs	7	7
Total fingerprinting time (hrs:mins)	∼08:39	∼04:51
Training time (hrs:mins)	∼01:05	∼0:43

**Table 3:** Default parameters values used in the evaluation.

Parameter	Range	Default
Learning rate	0.0001–0.2	0.001
Number of hidden neurons	20–1000	300
Batch size	1-Dataset size	128
Number of layers	1–30	3
Early Stopping Patience (epochs)	1–10	40
Number of samples per reference point	20–100	100
Number of epochs	Automatic by Early stopping	
Used devices	Google Pixel XL, Google Pixel 2XL	
Number of users	3	
Update rate (scan/sec)	2	

**Table 4:** The localization error percentiles in Office testbed.

Technique	Average	25th	50th	75th	Maximum
		Percentile	Percentile	Percentile	
*RRLoc*	**0.51 m**	**0.19 m**	**0.32 m**	**0.79 m**	**1.70 m**
*WiNar* [14]	0.89 m (−72%)	0.34 m (−82%)	0.73 m (−129%)	1.20 m (−53%)	2.99 m (−76%)
*WiDeep* [29]	1.46 m (−183%)	0.58 m (−208%)	1.17 m (−267%)	1.97 m (−151%)	4.49 m (−164%)
*Ranging* [17]	2.59 m (−401%)	1.44 m (−664%)	2.34 m (−632%)	3.68 m (−368%)	4.92 m (−189%)

**Table 5:** The localization error percentilesin the Lab testbed.

Technique	Average	25th	50th	75th	Maximum
		Percentile	Percentile	Percentile	
*RRLoc*	**0.59 m**	**0.12 m**	**0.42 m**	**1.08 m**	**1.83 m**
*WiNar* [14]	0.99 m (−69%)	0.19 m (−51%)	0.61 m (−45%)	1.77 m (−63%)	3.0 m (−64%)
*WiDeep* [29]	1.92 m (−226%)	1.06 m (−753%)	1.84 m (−337%)	2.69 m (−149%)	6.00 m (−228%)
*Ranging* [17]	2.86 m (−384%)	1.46 m (−1077%)	2.51 m (−496%)	3.85 m (−255%)	7.38 m (−304%)

## Data Availability

Not applicable.
